# Poor-Law Institutions and Nurse-Training

**Published:** 1916-11-04

**Authors:** 


### THE MATRONS' AND SISTERS' DEPARTMENT

### POOR-LAW INSTITUTIONS AND NURSE-TRAINING.

A MEMORIAL to Nurse Cavell has been placed, upon the anniversary of her tragic and lamented death, in the nurses' quarters at the Shoreditch Infirmary. The fact that Miss Cavell had held a post at the infirmary bears out the fact, upon which we have frequently insisted, that Poor-Law institution nursing is no longer an isolated service, but one in which large numbers of nurses are trained for general nursing work. There is, moreover, frequent and free interchange between that service and other branches of nursing.

The all-important questions at the outset of a nurse's career are: At what class of institution, and at which particular institution of that class, she shall seek admission for training. The decisive selection, once made, is usually irrevocable. In making it the experience of friends is, of course, often valuable, and questions of means and locality have often to be taken into consideration. It may be useful to inquire what are the prospects, from the nurse's point of view, of the training offered in the Poor-Law hospitals or infirmaries.

These institutions, which have developed upon the basis of the old workhouse sick-wards, differ in some essentials from the voluntarily supported hospitals. Those of them which are controlled upon the most advanced and generous lines merit the title of State hospitals; the principal distinctions are that, as compared with the "voluntary" hospitals, a somewhat less acute average class of disease is treated, and the medical staff is relatively small, there being usually no consulting staff and even, in the smaller institutions, one resident medical officer only. There is no specialised staff of gynæcological or ophthalmic surgeons, anæsthetists, pathologists, etc. There is, of course, no medical school. The larger and better-organised nurse-training schools (with which we are here especially concerned) are those attached to the separate infirmaries, which are entirely under the control of a medical superintendent, and so are "separate" from the workhouse, whether situated adjacent to it or not. The buildings are usually modern, good, and well equipped, and the treatment of the patients is in accordance with general hospital practice.

The probationer has, therefore, all necessary clinical facilities for instruction. She does not usually at present receive the preliminary training before entering the wards which forms so useful a part of the teaching in many of our great hospitals, but works (for two months or so) on trial in the wards under the supervision and observation of trained ward sisters, upon whose satisfactory report of her conduct and promise largely depends her acceptance for training. The menial work which she is called upon to do is reduced to a minimum by the employment of wardmaids or scrubbers, and by the assistance given by convalescent patients, whose employment to help in any nursing work is studiously avoided. Class instruction is suitable and efficient; it is arranged and given by the medical staff in conjunction with the matron, her assistants, the home sister, etc., or even a sister specially appointed for, and limited to the duty of, teaching. The fact that these several senior members of the nursing staff have often been themselves trained at as many different schools tends to annul any risk of "getting into a groove." The instruction is usually simple, direct, and practical; and a sound elementary groundwork, well within the range of her understanding, is secured for the nurse. Test examinations are held and, when the time comes, the examination for certificate is conducted under a selected examiner not otherwise connected with the school.

(*To be continued.*)

